# The Efficacy of Thermal Mechanical Fractional Injury System for Facial Rejuvenation: Subjective, Objective and Image Analysis Study

**DOI:** 10.1111/jocd.70063

**Published:** 2025-02-17

**Authors:** Guy Erlich, Ron Skorochod, Edmond Sabo, Rishi Saigal, Eliran Dahan, Yoram Wolf

**Affiliations:** ^1^ Unit of Plastic Surgery Hillel Yaffe Medical Center Hadera Israel; ^2^ Technion‐Israel Institute of Technology Rapapport Faculty of Medicine Haifa Israel; ^3^ Mehuedet HMO Tel Aviv Israel; ^4^ Buckingham University Buckingham UK; ^5^ Department of Pathology Carmel Medical Center Haifa Israel; ^6^ Mercy Medical Center Roseburg Oregon USA; ^7^ Sourasky Medical Centre – Ichilov Tel‐Aviv Israel; ^8^ Tel‐Aviv University Tel‐Aviv Israel

**Keywords:** aesthetic, facial rejuvenation, Tixel, TMFI

## Abstract

**Background:**

Facial aging is the result of a number of factors, including the resorption of skeletal tissue, the atrophy of fat pads, and the laxity of connective tissue. These factors contribute to a reduction in facial volume and the development of skin changes. Thermal mechanical fractional injury (TMFI) represents a minimally invasive solution, stimulating collagen production and enhancing the delivery of topical formulations via epidermal microcraters.

**Aim:**

This study seeks to evaluate the effects of TMFI on skin quality and introduce quantifiable methods to assess improvements.

**Patients and Methods:**

Adult patients seeking facial rejuvenation underwent three TMFI sessions, with a one‐month interval between each session. The assessment of skin quality was conducted using the Scientific Assessment Scale of Skin Quality (SASSQ), which was completed by blinded experts. Patient satisfaction was evaluated using the FACEQ scale. Additionally, objective texture analysis was performed using the MAZDA program, which quantified surface irregularities.

**Results:**

The objective assessments demonstrated statistically significant improvements in the following parameters: roughness, wrinkles, pore size, elasticity, pigmentation, and erythema (*p* < 0.001). The FACE‐Q results demonstrated high patient satisfaction, with a response rate of 68.4%. The MAZDA analysis confirmed significant texture improvements across most facial zones, with the exception of the medial forehead.

**Conclusions:**

The findings of this study indicate that TMFI is an effective intervention for improving skin quality in a diverse range of patients. This study contributes to the existing literature on TMFI, which supports its use as a valuable tool in the field of skin rejuvenation.

## 1 Introduction

Facial aging is a complex, multifactorial, and dynamic process. While comparable alterations manifest in all individuals, the extent and rate of aging exhibit variability and are influenced by intrinsic and extrinsic factors [1].

In general, the aged appearance of the face is the result of a reduction in facial volume of the deeper layers. The underlying pathogenesis is characterized by skeletal resorption and deep fat pad atrophy, as well as increased tissue laxity due to the weakening of retinacula cutis and thinning of the epidermis [2, 3, 4, 5].

To achieve skin rejuvenation, it is essential to facilitate new collagen synthesis, thereby increasing skin thickness, improving its texture and firmness, and reducing its laxity. Neocollagenesis can be achieved through the repair of tissue following physical or mechanical trauma, such as that caused by micro‐needling, heat damage (thermal mechanical fractional injury (TMFI)), or the introduction of foreign materials that triggers an immunological response (such as poly‐l‐lactic acid, PLLA) [3, 6].

TMFI is a novel strategy for skin rejuvenation and drug delivery. The procedure revolves around the transfer of direct heat through nonablative conduction, using brief contact of pyramidal tips that exert pressure on the skin, creating epidermal microcraters. The thermal effect of TMFI extends along the epidermis and superficial dermis, encouraging fibroblast proliferation and collagen production [7, 8, 9, 10, 11].

TMFI employs its technology to create transepidermal channels in the skin, enhancing the penetration of topically applied formulations. Its distinctive method produces observable channels in the skin that can remain open for over 24 h, enabling sustained delivery of drugs into the epidermis and superficial dermis [12, 13].

The procedure is generally well tolerated, with most patients reporting minimal discomfort and a low requirement for anesthesia. The reported downtime is minimal, often less than a day, and adverse events are rare, with only a few cases of hyperpigmentation observed, primarily in individuals with darker skin tones [14].

Overall, TMFI represents a valuable and versatile tool in dermatology with promising results for both esthetic and therapeutic applications.

In this study, we aim to further investigate the effect of TMFI technology on skin quality and introduce novel quantifiable methods to estimate the magnitude of change.

## 2 Materials and Methods

Nineteen adult patients who sought facial rejuvenation procedures at the clinic of the primary investigator were eligible for inclusion in the study.

Patients were included in the study if they had undergone treatment with TMFI and were between the ages of 18 and 65 years.

Patients were excluded from the study in instances of concomitant facial esthetic procedures in the month prior to the study or a lack of willingness to adhere to the prescribed treatment timeline and follow‐up schedule.

Prior to the commencement of treatment, all patients were required to sign an informed consent form indicating their willingness to participate in the study.

All patients were treated on three occasions, with a one‐month interval between each course of treatment. Photographic documentation was conducted at the baseline and one month following the final treatment session.

In order to evaluate the impact of TMFI treatment on skin quality, a number of different methods have been employed in this study.

First, an objective evaluation and ranking of skin quality based on images taken prior to treatment and one month following the last treatment session was conducted using the Scientific Assessment Scale of Skin Quality (SASSQ). Three independent esthetic experts were requested to complete the questionnaire based on the two provided images.

Experts were blinded to which image was of the patient before the treatment and which was of the latter.

Second, a subjective evaluation of patient satisfaction was conducted at the final follow‐up appointment using the FACEQ questionnaire.

**FIGURE 1:**
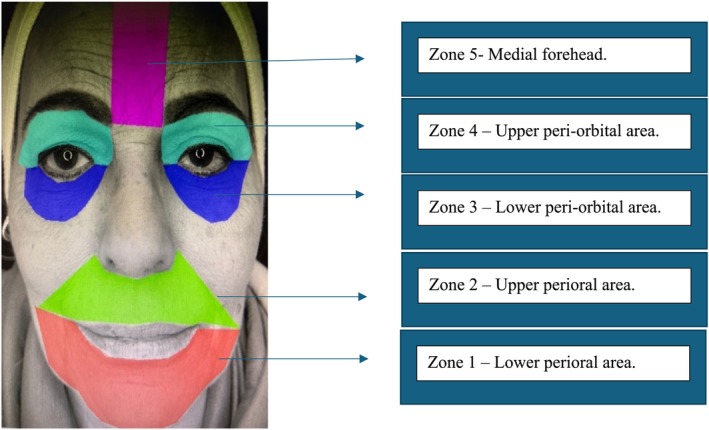
Visual representation of the corresponding facial areas analyzed using the MAZDA image analysis software.

Third, image analysis was conducted using the MAZDA program to evaluate skin texture at five facial zones for the baseline image and the follow‐up image. The facial zones are illustrated in Figure 1.

The MAZDA program assesses skin quality by quantifying pixel intensity variations across the skin surface in different directions. These parameters quantify the uniformity, consistency, and smoothness of the skin, facilitating objective confirmation of changes in these parameters following treatments. High pixel intensity variations in the images, which are depicted as lower “sum of averages,” correspond to a significant amount of surface irregularities, such as wrinkles, rough patches, and uneven skin texture.

Various image parameteres are represented at several axes; horizontal, vertical, and diagonal. The same parameters were compared before and after the treatments in the corresponding axis, and the difference was anlayzed using the student's *t* test to quantify the statistical significance of the difference. For example, the texture parameter at the horizontal axis is marked as S(1,0), the vertical is S(0,1), and the diagonal is S(1,1).

## 3 Results

In total, 19 patients were included in the study. However, 6 patients refrained from answering the FACE‐Q questionnaire following the last treatment session, accounting for a response rate of 68.4%.

Analysis of objective assessment conducted by three independent reviewers demonstrated consistent statically significant differences in the domains of “Roughness” (*p* < 0.001, consistently), “Wrinkles” (*p* < 0.001, consistently), and “Port size” (*p* < 0.001, *p* < 0.001, and *p* = 0.04). Additionally, “Elasticity” (*p* < 0.001, consistently), “Pigmentation” (*p* < 0.001, consistently), and “Erythema” (*p* = 0.08. 0.004, accordingly) were found to be statistically improved, per reviewers 1 and 2.

Table 1 demonstrates results across all domains segregated individually by reviewers. Results are represented as mean and standard deviation.

**TABLE 1:** SASSQ results across all domains segreated individually by reviewers.

Variable	Reviewer 1			Reviewer 2			Reviewer 3		
	Before mean, SD	After mean, SD	*p*	Before mean, SD	After mean, SD	*p*	Before mean, SD	After mean, SD	*p*
Elasticity	2.1, 1.4	1.6, 1.4	**< 0.001**	2.2, 1.3	1.7, 1.4	**< 0.001**	0.9, 1	1, 0.9	0.16
Wrinkles	2.1, 1.4	1.8, 1.3	**< 0.001**	2.1, 1.4	1.8, 1.2	**< 0.001**	1.3, 1	1.3, 1.2	**0.001**
Roughness	2.2, 1.4	1.8, 1.2	**< 0.001**	2.1, 1.3	1.6, 1.3	**< 0.001**	0.7, 0.8	0.7, 0.7	**< 0.001**
Pigmentation	2.7, 1.1	2.4, 1.2	**< 0.001**	2.6, 1.1	2.4, 1.3	**< 0.001**	1.4, 0.5	1.1, 0.7	0.924
Erythema	2.1, 1.2	2.1, 1.3	**0.008**	2, 1.3	2.1, 1.3	**0.004**	1.1, 1	1.7, 1.5	0.81
Blemishes	3.1, 1	2.8, 1	**0.004**	3, 1	2.7, 1	0.05	3,1	3,1	1
Port size	2.2, 1.2	2.1, 1.3	**< 0.001**	2.3, 1.1	2.1, 1.4	**< 0.001**	0.8, 0.6	0.7, 0.6	**0.036**

*Note:* Results are represented as mean and standard deviation. Bold signifies statistical significance at p value < 0.05.

Analysis of FACE‐Q responses demonstrated a mean overall result of 20.5 with a standard deviation of 4.1, with the following component distribution:

Mean, Standard deviation for the domain “I am pleased with the result” was 3.6 ± 0.6.

Mean, Standard deviation for the domain “The result turned out great” was 3.5 ± 0.7.

Mean, Standard deviation for the domain “The result was just as I expected” was 3.3 ± 0.8.

Mean, Standard deviation for the domain “I am surprised at how good I look in the mirror” was 3.4 ± 0.8.

Mean, Standard deviation for the domain “The result is fantastic” was 3.4 ± 0.8.

Mean, Standard deviation for the domain “The result is miraculous” was 3.3 ± 0.9.

Table 2 demonstrates results across all domains. Results are represented as mean and standard deviation.

**TABLE 2:** FACE‐Q results across all domains.

Patient (*N*)	Domain						SUM
	A	B	C	D	E	F	
1	4	4	4	4	4	4	24
2	4	4	4	4	4	4	24
4	3	3	2	3	3	2	16
6	4	3	3	3	3	3	19
7	4	4	4	4	4	3	23
8	4	4	4	4	4	4	24
12	4	4	4	4	4	4	24
13	2	2	2	2	2	2	12
14	4	3	3	4	3	4	21
15	4	4	4	4	4	4	24
17	4	4	3	4	4	4	23
18	4	4	4	3	4	4	23
19	3	3	3	2	2	2	15
Mean, SD	3.6 ± 0.6	3.5 ± 0.7	3.3 ± 0.8	3.4 ± 0.8	3.4 ± 0.8	3.3 ± 0.9	20.5 ± 4.1

*Note:* Results are represented as mean and standard deviation.

The analysis of MAZDA image analysis results indicates a statistically significant improvement in the “sum of averages” across all parameters and axes, with the exception of facial zone 5, which represents the medial forehead and consistently demonstrated a lack of statistical significance.

Table 3 demonstrates results across all parameters and axes. Results are represented as average before and after with the corresponding *p* value.

**TABLE 3:** MAZDA image analysis results across all parameters and axises.

Parameter	Zone	Average before	Average after	Change	*p*
S(1,0)	Zone 1	78.91	84.191	5.281	0.004
	Zone 2	84.799	90.651	5.852	0.0002
	Zone 3	81.378	84.843	3.465	0.033
	Zone 4	67.996	74.447	6.451	0.0007
	Zone 5	95.47	99.409	3.939	0.0573
S(0,1)	Zone 1	78.915	84.198	5.283	0.004
	Zone 2	84.818	90.669	5.851	0.0002
	Zone 3	81.386	84.853	3.467	0.0338
	Zone 4	68.003	74.446	6.443	0.0007
	Zone 5	95.472	99.413	3.941	0.0573
S(1)	Zone 1	78.915	84.198	5.283	0.004
	Zone 2	84.821	90.674	5.853	0.0002
	Zone 3	81.393	84.862	3.469	0.0337
	Zone 4	68.009	74.457	6.448	0.0007
	Zone 5	95.479	99.419	3.94	0.0573
S(1,‐1)	Zone 1	78.924	84.211	5.287	0.004
	Zone 2	84.817	90.666	5.849	0.0002
	Zone 3	81.389	84.856	3.467	0.0337
	Zone 4	68.008	74.452	6.444	0.0007
	Zone 5	95.478	99.419	3.941	0.05734
S(2,0)	Zone 1	78.93	84.215	5.285	0.004
	Zone 2	84.81	90.662	5.852	0.0002
	Zone 3	81.387	84.856	3.469	0.0337
	Zone 4	68.019	74.47	6.451	0.0007
	Zone 5	95.477	99.416	3.939	0.0574
S(0,2)	Zone 1	78.941	84.231	5.29	0.004
	Zone 2	84.847	90.697	5.85	0.0002
	Zone 3	81.404	84.875	3.471	0.0337
	Zone 4	68.031	74.467	6.436	0.0007
	Zone 5	95.481	99.423	3.942	0.0572
S(2)	Zone 1	78.941	84.229	5.288	0.004
	Zone 2	84.853	90.706	5.853	0.0002
	Zone 3	81.417	84.892	3.475	0.0337
	Zone 4	68.044	74.49	6.446	0.0007
	Zone 5	95.494	99.435	3.941	0.0573
S(2,‐2)	Zone 1	78.958	84.253	5.295	0.004
	Zone 2	84.846	90.691	5.845	0.0002
	Zone 3	81.409	84.88	3.471	0.0336
	Zone 4	68.04	74.479	6.439	0.0007
	Zone 5	95.493	99.434	3.941	0.0573
S(3,0)	Zone 1	78.949	84.239	5.29	0.004
	Zone 2	84.82	90.672	5.852	0.0002
	Zone 3	81.396	84.869	3.473	0.0336
	Zone 4	68.041	74.493	6.452	0.0007
	Zone 5	95.483	99.422	3.939	0.0574
S(0,3)	Zone 1	78.966	84.262	5.296	0.004
	Zone 2	84.875	90.724	5.849	0.0002
	Zone 3	81.42	84.895	3.475	0.0337
	Zone 4	68.059	74.489	6.43	0.0007
	Zone 5	95.49	99.433	3.943	0.0572
S(3)	Zone 1	78.965	84.258	5.293	0.004
	Zone 2	84.883	90.738	5.855	0.0002
	Zone 3	81.44	84.92	3.48	0.0336
	Zone 4	68.08	74.523	6.443	0.0007
	Zone 5	95.508	99.45	3.942	0.0573
S(3,‐3)	Zone 1	78.99	84.295	5.305	0.004
	Zone 2	84.873	90.715	5.842	0.0002
	Zone 3	81.428	84.903	3.475	0.0335
	Zone 4	68.071	74.505	6.434	0.0007
	Zone 5	95.506	99.45	3.944	0.0573
S(4,0)	Zone 1	78.968	84.261	5.293	0.004
	Zone 2	84.831	90.683	5.852	0.0002
	Zone 3	81.405	84.882	3.477	0.0335
	Zone 4	68.062	74.517	6.455	0.0007
	Zone 5	95.489	99.429	3.94	0.0574
S(0,4)	Zone 1	78.991	84.293	5.302	0.004
	Zone 2	84.902	90.75	5.848	0.0002
	Zone 3	81.436	84.915	3.479	0.0336
	Zone 4	68.087	74.51	6.423	0.0007
	Zone 5	95.498	99.442	3.944	0.05746
S(4)	Zone 1	78.989	84.286	5.297	0.004
	Zone 2	84.913	90.768	5.855	0.0002
	Zone 3	81.462	84.949	3.487	0.0335
	Zone 4	68.115	74.556	6.441	0.0007
	Zone 5	95.521	99.466	3.945	0.0573
S(4,‐4)	Zone 1	79.022	84.335	5.313	0.004
	Zone 2	84.899	90.737	5.838	0.0002
	Zone 3	81.446	84.926	3.48	0.0334
	Zone 4	68.101	74.53	6.429	0.0007
	Zone 5	95.519	99.464	3.945	0.0572
S(5,0)	Zone 1	78.987	84.284	5.297	0.004
	Zone 2	84.841	90.693	5.852	0.0002
	Zone 3	81.414	84.895	3.481	0.0334
	Zone 4	68.084	74.54	6.456	0.0007
	Zone 5	95.495	99.436	3.941	0.0574
S(0,5)	Zone 1	79.016	84.323	5.307	0.004
	Zone 2	84.928	90.775	5.847	0.0002
	Zone 3	81.452	84.935	3.483	0.0335
	Zone 4	68.114	74.532	6.418	0.0007
	Zone 5	95.506	99.451	3.945	0.0572
S(5)	Zone 1	79.012	84.314	5.302	0.004
	Zone 2	84.941	90.798	5.857	0.0002
	Zone 3	81.483	84.976	3.493	0.0333
	Zone 4	68.15	74.59	6.44	0.0007
	Zone 5	95.534	99.48	3.946	0.0573
S(5,‐5)	Zone 1	79.053	84.374	5.321	0.004
	Zone 2	84.924	90.758	5.834	0.0002
	Zone 3	81.463	84.95	3.487	0.0332
	Zone 4	68.129	74.554	6.425	0.0007
	Zone 5	95.532	99.479	3.947	0.0572

*Note:* Results are represented as average before and after with the corresponding *p* value.

## 4 Discussion

TMFI technology has been found to be highly efficient in the treatment of actinic keratosis, hypertrophic, or keloid scars and facial rejuvenation. In addition to showcasing promising esthetic outcomes, the procedure‐related pain, discomfort, and work leave time are minimal and well tolerated [9, 13, 15, 16, 17, 18].

Artzi et al. found that topical triamcinolone acetonide and 5‐fluorouracil delivery to elevated scars using the technology, significantly approved their appearance with high satisfaction reported by patients [15].

Oren‐Shabtai et al. sought to evaluate the efficacy of TMFI on the reduction of actinic keratosis (AK) lesions in a prospective study. All patients underwent several treatment rounds with the device several weeks apart.

A number of lesions after the last treatment session were found to significantly decrease, all while demonstrating a high level of patient satisfaction from the proccess and the esthetic result [17].

In the esthetic facial rejuvenation scope, Elman et al. demonstrated that esthetic treatments with TMFI in 26 subjects resulted in high patient satisfaction and wrinkle attenuation, with minimal treatment‐related pain and down time [9].

Daniely et al. performed a retrospective chart review of 24 patients whom underwent a series of treatments with TMFI for facial skin rejuvenation. The authors sought the aid of independent reviewers and asked them to rate the difference between standardized pre and post‐procedural images on several parameters. The authors found that on all queried parameters, an overall improvement was noted [8].

In our study, we found conclusive evidence supporting the beneficiary qualities of a thermal fractional skin rejuvenation system. Subjective, as well as objective measures, unanimously demonstrated improvement in various aspects of skin quality.

Patients reported high satisfaction rates with the esthetic results, despite homogenous baseline characteristics. These findings were further supported by independent reviewers evaluating their images before and after the last procedure.

The results were further quantified using image analysis techniques to allow for unbiased and quantifiable conclusions.

An interesting point that should be mentioned is that image analysis was the only modality that took into consideration changes in independent facial areas. As a result, we found that TMFI is less efficient in improving skin quality in the medial forehead compared to other facial areas.

This conclusion demonstrated the unique and important potential of image analysis. Artificial intelligence and machine learning bear the potential to note differences that are often unrecognized by the naked eye.

Utilization of image analysis modalities in the quantification of effect size allows for robust and reproducible evidence that is independent of patient or operator‐related confounders.

Although image analysis is often hard to mathematically comprehend, the results produced by the software allow for undisputable evidence that is not influenced by external factors.

Despite the evident advantages, it is important to state that quality image analysis is dependent on experienced data analysts and researchers. Methodical impurities can significantly impair conclusions and therefore must be interoperated with caution.

Although the results of our study are based on subjective and objective evidence, we must mention the limitations that are associated with it.

First, the response rate of patients to the FACE‐Q questionnaire was approximately 70%. Despite being high and clinically acceptable, it is crucial to understand that the non‐responders who were lost to follow‐up could have skewed the results of our study, leading to a differential misclassification.

To overcome this potential bias, other modalities have been implemented to provide objective evidence.

Second, although an encompassing and rigorous research methodology was applied for the purpose of the study, the low sample size must be mentioned when interpreting our results.

In conclusion, our study found that TMFI improves skin quality in a wide range of patients. This study adds to the current literature that supports TMFI as a useful tool in the armamentarium of skin rejuvenation modalities.

Further research focusing on complementary modalities and the symbiotic effect should be carried out to improve patient outcomes.

## Author Contributions

G.E. and Y.W. conceived the protocol and study. G.E. performed the procedures. R.S., E.D. and E.D. assisted in the data analysis and image evaluation. G.E. and R.S. wrote and revised the research paper and Y.W. critically revised it.

## Conflicts of Interest

The authors declare no conflicts of interest.

## Data Availability

The data that support the findings of this study are available from the corresponding author upon reasonable request.
